# Levine Cancer Institute Financial Toxicity Tumor Board: A Potential Solution to an Emerging Problem

**DOI:** 10.1200/OP.21.00124

**Published:** 2021-06-08

**Authors:** Derek Raghavan, Nicole A. Keith, Hughes R. Warden, Seungjean Chai, Wendy Jo Turan, Jaynie Moroe, Donna Feild, Thomas Gregory Knight

**Affiliations:** ^1^ Levine Cancer Institute, Atrium Health, Charlotte, NC

## Abstract

**PURPOSE:**

Fiscal distress or “financial toxicity,” in which patients experience challenges in paying for treatment, are becoming dominant problems for patients with cancer because of burgeoning health care costs and strategies implemented by health insurance payers to reduce their level of expenditure. We report the structure and function of the first Financial Toxicity Tumor Board (FTTB). Modeled on the concept of a conventional multidisciplinary tumor board, FTTB functions as a multidisciplinary conference providing broad problem-solving approaches to financial toxicity.

**METHODS:**

The FTTB, with participation from physicians, nurses, financial counselors, nurse navigators, social workers, and administrators, meets monthly and is focused on financial toxicity and financial worry experienced by patients with cancer. It is linked to a Patient Assistance Program for oncologic pharmaceutical agents as this domain constitutes a critical area of financial toxicity for many patients.

**RESULTS:**

In the first years of function, more than $55-$60 million of personal expenditure has been avoided for 1,749 and 1,819 patients, respectively, as well as more than $1.3 million copay assistance provided for financially challenged patients. Problems addressed have included payer impediments, underinsurance, complexities of certification, coding or billing issues, and inadequate internal standard operating procedures.

**CONCLUSION:**

A focus on proactive management of financial toxicity through the function of multidisciplinary FTTBs substantially ameliorates this burgeoning international problem. This concept is presented early as it may be leveraged readily in other centers.

## INTRODUCTION

One of the greatest emerging challenges of modern health care is the burgeoning costs associated with the advances in diagnosis and management. Irrespective of whether care is provided by a nationalized medical system, private medicine, or an amalgam of the two, the costs of hospitalization, diagnostic and therapeutic procedures, and equipment and labor have increased dramatically in the past 30 years.^1,2^ Government is increasingly challenged in paying for the high-quality health care that is promised on the electoral hustings, and thus many strategies have evolved to control expenditure and to rationalize the choices offered and the provision of services.^3-6^

Despite the attention focused on this domain by patients, payers, physicians, and hospital and health care systems, patients increasingly list financial distress as a key problem in their care and view this as a major factor in their quality of life.^7,8^ The cost of oncologic care sits at the high end of this situation, inflated particularly by increasingly complex technology, the costs of pharmaceuticals, increased regulatory oversight, and documentation.^4-6^ The term financial toxicity was created to describe the total impact of this financial burden on patients with cancer and has been linked with bankruptcy, noncompliance to treatment, worsened outcomes, and increased mortality.^7-12^ There is a growing consensus on the need to move to an interventional paradigm to resolve this issue, but thus far research and/or implementation have primarily consisted of small pilot studies.^13-16^

The Levine Cancer Institute (LCI) was created to offer oncologic services for Atrium Health, a system composed of more than 40 hospitals and 900 offices that provides more than 12 million encounters per year in North Carolina, South Carolina, and Georgia. LCI sees more than 18,000 new cases per year and provides services for more than 200,000 visits annually and additionally provides more than 60,000 nonbilled interactions in outreach education and prevention activities. Like other major cancer centers, LCI has available all oncologic services, including surgery, radiation, chemotherapy, survivorship and palliative services, pain management, cancer clinical and translational trials, bone marrow transplantation, chimeric antigen receptorT-cell treatment for hematologic malignancies, cancer education, outreach, and screening.^17^ LCI has routinely used a clinical electronically accessible pathways system, which was created in collaboration with Accenture Inc (Charlotte, NC) and which provides standardized first- and second-line, evidence-based treatment algorithms for all patients at all 25 sites. This system integrates standard practice algorithms with the cancer trials menu, biospecimen protocols, and implementation of supportive oncology consultation, and thus creates a more standardized cost-effective treatment approach.^18^ Atrium Health is is a safety net organization and LCI was the first to develop a free mobile low-dose computed tomography scanning unit that has been successfully applied to underserved and underinsured populations.^19^ Treatment outcomes for underserved minority populations have also been shown to be equivalent to those of insured majority patients, demonstrating the provision of true equity in health care and outcomes.^19,20^

Thus, with a large population of patients requiring complex therapies, including a broad range of insured, underinsured, and uninsured patients, the spectrum and intensity of financial toxicity has been identified and characterized, and reflects the patterns seen nationally.^7,8^ Initially, this was managed in a piecemeal fashion, with individual physicians, nurses, or financial counselors requesting assistance from a range of social and financial support operations internally and externally. However, in 2019, analogous to the multidisciplinary tumor conferences that are the mainstay of cancer management in most major institutions, a Financial Toxicity Tumor Board (FTTB) was established as a regular cancer management entity, focused on addressing the financial problems of patients. As part of the focus on reducing financial toxicity, a complementary structure, the Patient Assistance Program (PAP) for oncologic pharmaceutical agents, had previously been rolled out across all LCI sites and had helped to define the needs leading to the establishment of the FTTB.

We report the establishment, structure, function, and preliminary outcomes of the LCI FTTB as a potential model that may help to reduce the impact of burgeoning costs and charges in other medical facilities, not necessarily restricted to the domain of oncology practice.

## METHODS

### Establishment and Structure

In September 2019, the FTTB was established as a multidisciplinary scheduled conference monthly, with the intention of addressing complex fiscal issues, identifying frequent or repeated problems that required changes of standard operating procedures (SOPs), and to allow detailed discussion of cases that were not being effectively handled by standard patient and financial support operations. FTTB was designed to involve participants from several different domains within the Institute (Table 1). Specific participants included physicians, nurses, nursing and medical administrators, the LCI chief financial officer and members of his staff, financial counselors, social workers, nurse navigators, and oncology pharmacy personnel. Because of the perceived sensitive, confidential, and personal nature of fiscal status, as repeatedly reported in electronic surveys by our patients, and after consideration by the Patient Family Advisory Council, we omitted patient representatives from the initial FTTB, although this decision remains under review.

**TABLE 1.**
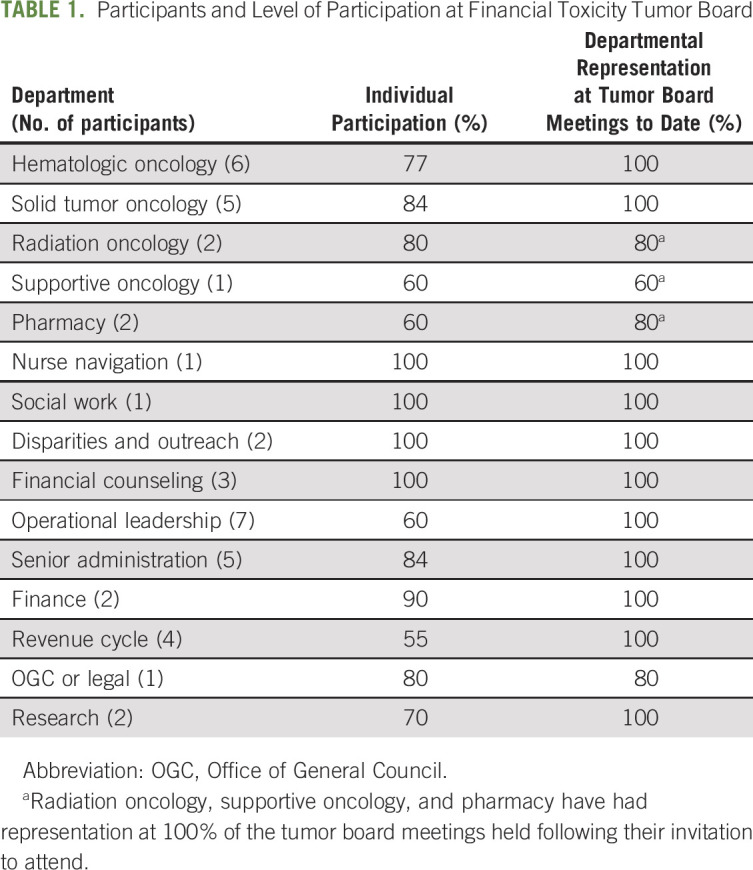
Participants and Level of Participation at Financial Toxicity Tumor Board

It is noteworthy that the Director of Nurse Navigation was routinely present, representing a team of 30 nurse navigators. The Director of Financial Counseling was also a routine participant, representing a team of eight financial counselors, providing an effective mechanism for translation of information and new SOPs from FTTB to relevant support staff.

The meetings were initially scheduled every 4 weeks, but with the expectation that they would eventually be required to occur more frequently. A quorum required at least one representative from each of the cited departments and disciplines. The meetings were structured, based on a precirculated agenda, which included identification of problems, specific illustrative cases, discussions of the outcomes of previous cases, and detailed attention to paradigm problems and evolution of SOPs to address repeated significant problems (as listed in Table 2).

**TABLE 2.**
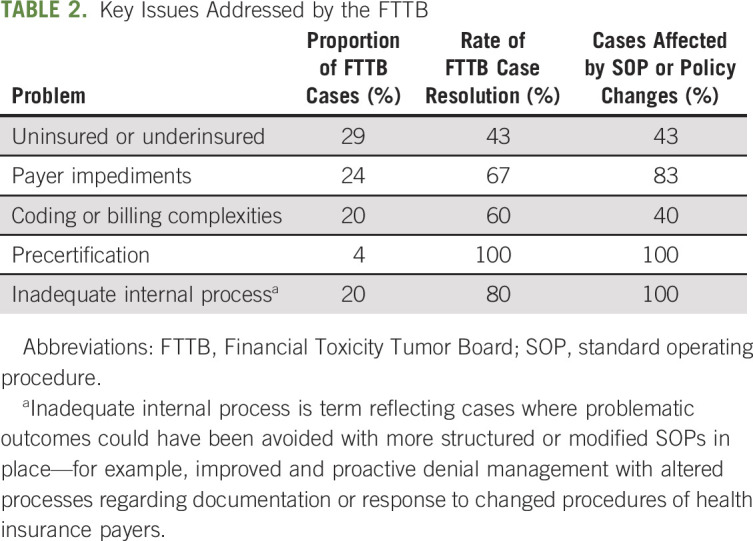
Key Issues Addressed by the FTTB

The establishment of the FTTB was advertised within the LCI, with information directed to all patients, caregivers, and staff. Signage was posted throughout the branches of the institute, and relevant patient-facing staff were instructed to alert patients to the availability of this support service. A document introducing the concept of FTTB and soliciting cases was e-mailed to all leaders and managers within LCI. As noted above, key patient support teams as well as a broad representation of clinical staff were regular participants to ensure strong linkage to patients with potential concerns.

To increase the potential for specific financial or insurance issues to be referred for FTTB discussion, access for referral was available to patients, family members or caregivers, physicians, nurses, social workers, financial counselors, oncology pharmacists, front-line clerical and support staff, and members of the administration. Cases were triaged by members of the Finance Department or financial counselors based on acuity and severity: if they were simple items that could be handled operationally (eg, failure to secure Medicaid or Medicare coverage, lack of documentation, or reluctance to disclose financial issues) by financial counselors, members of the Finance Department, a clinician, nurse navigator, or a social worker would manage the problem; if they represented more complex issues or operational lesions, they were referred for assessment and management by the multidisciplinary FTTB. In addition, the Tridiuum patient assessment electronic tool (Tridiuum, Philadelphia, PA), which includes a screening question on financial toxicity, has been routinely used and has led to additional referrals.

### PAP for Pharmaceutical Agents

In conjunction with the perceived need for the FTTB, a linked initiative was the expansion of the PAP for injectable pharmaceutical agents and copay assistance as a conjoint initiative between the LCI and the Atrium Health Department of Pharmacy. Under the direction of the Vice President of LCI Pharmaceutical Services, this initiative was designed to (1) manage proactively the availability of free cytotoxic, symptom management, and targeted agents; (2) proactively manage copay assistance for financially challenged patients with defined criteria; and (3) manage bad debts associated with lack of insurance to cover those complex medications. It became clear that considerable nursing time was being expended in addressing these issues; initially a nursing full-time equivalent role was converted into a designated pharmacy technician in an effort to focus the required skill sets in a more directed fashion. Initial success and improved patient satisfaction and rapidly improved patient savings led to specific pharmacy technicians being recruited to cover LCI with a primary responsibility to capture and manage the above issues proactively, thus reducing stress for financially challenged patients. A systematic approach toward repeated review of need for each new patient was implemented. As part of this strategy, all new infusions and regimen changes were evaluated for eligibility for free drug programs or copay assistance. A team of pharmacy technicians or financial coordinators is responsible for reordering medications before administration, linked to review of the financial and insurance status of the involved patients, and ensuring accurate accounting and linkage of replacement drugs to the relevant patients.

Another tool implemented to address financial toxicity was the physical or virtual embedding of pharmacy technicians within physician office practices. The purpose of these pharmacy technicians was to track and coordinate expensive oral medication needs for patients. Previous authorizations, copay assistance, free drug procurement, and directing to appropriate specialty pharmacies based upon a patient's insurance are handled by this group. As an additional function, an algorithm was developed wherein a pharmacy technician performed an additional check to ensure that previous authorizations were in place 72 hours in advance of scheduled infusions for all drugs, thus reducing unnecessary insurance challenges. LCI has adopted the use of biosimilars in an effort to reduce costs to payers and patients, although we mandate a meticulous review by the Pharmaceutics and Experimental Therapy Committee of the efficacy and toxicity data of any proposed biosimilar before acceptance into the formulary.

## RESULTS

### FTTB

After a year of operation, patterns of the dominant and most frequent presenting problems began to emerge (Table 2). Although our approach was no complete panacea for fiscal concerns and financial toxicity, given that problems continued to occur, we were able to show that FTTB addressed the majority of issues, reduced patient debt and distress, and also reduced the frequency of the most common presenting concerns.

As noted in Table 2, the majority of identified problems have fallen into broad categories, with the most common being a lack of adequate health insurance coverage (either complete or partial), impediments to reimbursement on the part of insurers (disputes over the medical necessity of treatment, evolving complexities in the precertification and claims submission processes, changing requirements for medical documentation, and altered internal procedures), and inadequate internal guidelines for communication between clinical and nonclinical teams. Additionally, repeated cases highlighted problems with health literacy, financial accessibility of novel therapeutics, and costs associated with complex surgical or radiation procedures. In some instances, lack of awareness by clinical staff of the fiscal consequences of selection of treatment, unplanned diagnostic tests, or treatments without optimal prior authorization has been identified and the issues formally circulated to the clinical staff to avoid repetition.

### Typical Case Study

One of the earliest cases shared with the FTTB was that of a 64-year-old patient undergoing adjuvant chemotherapy following surgical treatment of a pancreatic tumor. Upon receipt of the patient's planned course of treatment, the insurance company advised that no precertification was required, and the patient then received several cycles of chemotherapy. Months after treatment began, the patient received notice that the chemotherapy claims had been denied by the insurer, who indicated that a required precertification was never obtained. Following a review of the denied claims, it was noted that one drug in the treatment regimen required precertification through the patient's pharmacy benefits, rather than the medical plan, which was outside the norm for outpatient chemotherapy. Per instructions from the Explanation of Benefits issued by the patient's insurer, the patient was billed nearly $42,000 for the denied medication.

This case was presented to the tumor board after the patient requested assistance from our financial counseling team. The financial counselor was able to secure a retroactive and ongoing precertification for the entire course of treatment, effectively eliminating the patient's financial responsibility. Additionally, to avoid similar denials in the future, a process was implemented whereby our representatives now specifically confirm that no part of a patient's treatment requires a separate precertification through their specialty pharmacy benefits.

### Patient Assistance Program

Table 3 illustrates the impact of the first 2 years of the PAP. The PAP served 1,749 and 1,819 patients, respectively, in 2019 and 2020, with a financial savings of $55-$60 million in each of these years.

**TABLE 3.**
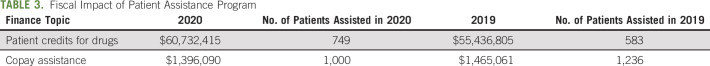
Fiscal Impact of Patient Assistance Program

## DISCUSSION

The regular attendance at the LCI FTTB by a high percentage of participants over a prolonged period suggests the acceptance and feasibility of this concept and the potential for adapting it to other clinical settings. During the COVID-19 pandemic, we have adapted many of our clinical strategies to increase the level of safety for patients and staff^19^ and have moved our multidisciplinary tumor conferences to virtual platforms. We have also done this for the FTTB, without loss of attendance or the ability to carry out all its functions, and it may be that this conference will be maintained as a virtual meeting when the COVID-19 acute threat has subsided.

The clustering of fiscal problems suggests that health care systems could potentially alleviate many of the issues facing patients by proactively establishing SOPs that address the most prevalent causes of financial toxicity while also ensuring that these procedures are familiar to staff and also communicated clearly to patients and caregivers. The Case History indicates both the magnitude and scale of this problem; in this example, because of the substantial level of pharmaceutical cost, an underinsured patient was saved more than $40,000 of personal expenditure. Given that the FTTB and its component services has addressed the needs of more than 1,500 patients per year for the past 2 years, it is not surprising that patient-related savings of more than $55 million per year have occurred, especially when one considers that the costs of completed treatment programs of chimeric antigen receptor T-cell therapy and some of the newer immune-oncology and targeted agents can exceed $300,000-$500,000, and prolonged in-patient stays after complicated surgery with intensive care unit management can also accrue similar patient costs for uninsured or underinsured patients.

We anticipate moving to a biweekly format in the next months to accommodate the increasing number of cases of financial distress in patients with cancer associated with the financial implications of the pandemic—specifically unemployment, loss of insurance, and expenses associated with hospital and intensive care unit admissions and expensive antiviral and other medications. Although our structured approach to pandemic cancer management has limited the numbers of COVID-positive patients with cancer,^19^ we anticipate increasing numbers requiring FTTB assistance as a reflection of general community exposure and the associated economic implications.

The experience of patients with cancer with financial toxicity is certainly not unique, and these principles could easily be applied to patients with any diseases associated with substantial adverse financial impact. Given that it is well documented that fiscal toxicity is associated with worse outcomes (including more advanced stage of presentation and consequently increased costs of care, increased morbidity, and higher mortality), it is logical that effective and proactive management of this issue would be more cost-effective for the provision of medical care and would enhance health equity.
